# Soil pH Mapping with an On-The-Go Sensor

**DOI:** 10.3390/s110100573

**Published:** 2011-01-07

**Authors:** Michael Schirrmann, Robin Gebbers, Eckart Kramer, Jan Seidel

**Affiliations:** 1 Leibniz-Institute for Agricultural Engineering, Department of Engineering for Crop Production, Max-Eyth-Allee 100, D-14469 Potsdam, Germany; E-Mail: rgebbers@atb-potsdam.de; 2 HNEE School for Sustainable Development Eberswalde, Friedrich-Ebert-Str. 28, D-16225 Eberswalde, Germany; E-Mail: Eckart.Kramer@hnee.de; 3 Technical University of Dresden, D-01062 Dresden, Germany; E-Mail: seidel_j@gmx.de

**Keywords:** precision agriculture, soil sensors, digital soil mapping, soil sampling, pH, antimony electrode, Veris pH Manager™

## Abstract

Soil pH is a key parameter for crop productivity, therefore, its spatial variation should be adequately addressed to improve precision management decisions. Recently, the Veris pH Manager™, a sensor for high-resolution mapping of soil pH at the field scale, has been made commercially available in the US. While driving over the field, soil pH is measured on-the-go directly within the soil by ion selective antimony electrodes. The aim of this study was to evaluate the Veris pH Manager™ under farming conditions in Germany. Sensor readings were compared with data obtained by standard protocols of soil pH assessment. Experiments took place under different scenarios: (a) controlled tests in the lab, (b) semicontrolled test on transects in a stop-and-go mode, and (c) tests under practical conditions in the field with the sensor working in its typical on-the-go mode. Accuracy issues, problems, options, and potential benefits of the Veris pH Manager™ were addressed. The tests demonstrated a high degree of linearity between standard laboratory values and sensor readings. Under practical conditions in the field (scenario c), the measure of fit (r^2^) for the regression between the on-the-go measurements and the reference data was 0.71, 0.63, and 0.84, respectively. Field-specific calibration was necessary to reduce systematic errors. Accuracy of the on-the-go maps was considerably higher compared with the pH maps obtained by following the standard protocols, and the error in calculating lime requirements was reduced by about one half. However, the system showed some weaknesses due to blockage by residual straw and weed roots. If these problems were solved, the on-the-go sensor investigated here could be an efficient alternative to standard sampling protocols as a basis for liming in Germany.

## 1. Introduction

The pH value is the negative logarithm of the molar concentration of protons (or hydronium-ions) in a solution:

(1) $$pH=-\text{log}\;\left(a_{H^{+}}\right)\;\left\lfloor \text{mol}\cdot L^{-1}\right\rfloor$$

As a measure of soil acidity or alkalinity, soil pH constitutes one of the most important chemical soil parameters [1]. Generally, soil pH values outside the range of 5.5 to 6.5 are considered as nonoptimum because they can have negative impacts on nutrient availability [2,3], soil structure [4], soil organisms [5], and can make plants more sensitive to diseases [6]. Due to uptake by plants and natural leaching of alkaline soil compounds, acidification is common among soils in temperate climates. Fewer soils, like soils on limestone or on glacial till, have high pH values. The regulation of soil pH by applying alkaline or acid fertilizers can limit effects of extreme acidic or alkaline soil conditions, which in turn improves crop production and resource efficiency [7]. In a global view, soil pH regulation becomes a vital strategy within a radically changing world, where globally average crop yields must be increased by 60% to 120% until 2050 to meet the needs of the human population and dietary habits [8].

It is well known that pH values, and consequently liming requirements, can vary within an agricultural field. Thus, it may be profitable to apply lime according to the spatial variation of soil pH within the fields using precision agriculture (PA) technologies [9–11]. This assumes that accurate maps of soil pH are used as inputs. The quality of pH maps is predominantly influenced by sampling density, while other factors such as measurement errors are less important [12].

It turns out that sufficient sampling density can be relatively high. Mueller *et al.* analyzed different sampling resolutions (30–100 m) and concluded that grid sampling at 100 m sample distance is grossly inadequate to resolve within-field variability of soil pH [13]. Likewise, Brouder *et al.* found that sample distances greater than 100 m cannot reveal the spatial pattern of soil pH and are too uninformative for map generation [14]. Nevertheless, current standard sampling strategies do not allow for a density beyond 1 sample ha^−1^ because it involves manual soil sampling and laboratory analysis, which is costly and time consuming [15,16].

**Figure 1.**
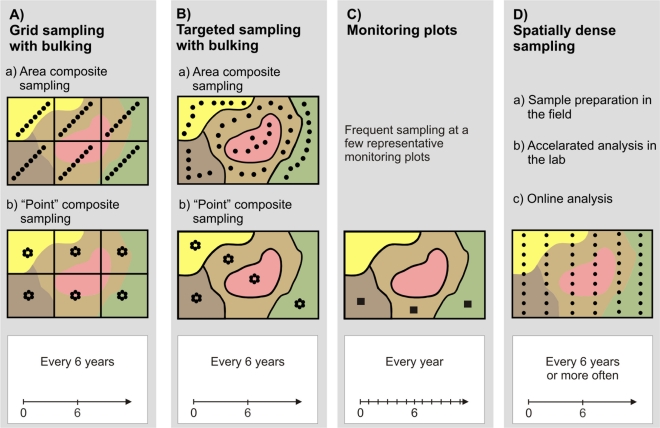
Sketch of standard and alternative sampling strategies for soil pH mapping (details are given in the text).

Standard and alternative sampling procedures are summarized in Figure 1. Area (or cell) sampling on a regular grid with bulking of several subsamples into one composite sample (Figure 1A) is the most common strategy and is often recommended by authorities like the German Advisory Board for Agricultural Analytics, VDLUFA [17], or the British Ministry of Agriculture, Fisheries and Food [18]. According to the guidelines in [17], fields should be subdivided into regular parcels of 3 to 5 ha. Within these parcels, 15 to 20 subsamples should form one composite sample. The subsamples should be taken evenly distributed on a diagonal or Z-shaped track (Figure 1A-a). Bulking increases the sampling support and improves the precision when average values are required [19]. However, grid area composite sampling may not reveal spatial variation very well because soil properties are attributed to arbitrarily chosen zones that may not coincide with the spatial pattern observed in the field.

Point composite sampling on a regular grid (Figure 1A-b) is based on the bulking of samples taken within a few meters around a (regular) grid point [17,20]. Contrary to grid area composite sampling, this kind of sampling allows for interpolation, which can generate smooth surfaces for soil properties, like pH. However, care should be taken because interpolation is only sensible when spatial dependency between the pH values is evident. Thus, VDLUFA suggests using grid point composite sampling only with sampling densities less than 3 ha [17].

Targeted (directed or zone) sampling (Figure 1B) takes into account previous knowledge about the distribution of the soil property of interest within a field [20–22]. This information may come from preceding intensive soil surveys or other auxiliary data (e.g., aerial images, terrain models). Based on these ancillary data, the field is subdivided into irregular shaped zones, which are addressed either by area composite (Figure 1B-a) or by point composite sampling (Figure 1B-b). Targeted sampling relies on the correlation (coincidence) between the ancillary data and the soil parameter under consideration (the pH distribution in our case). This is not always easy to establish [23]. Thus, it cannot be said that targeted sampling is generally better than grid sampling [21].

The use of monitoring plots makes even stronger assumptions about the representativeness of sampling locations (Figure 1C). It is assumed that a small plot of a few square meters is representative for the dominant soil types within the field and that the rest of the field will respond similarly to the monitoring area [17,24]. Only a very few number of samples are used which allows the increase of samples over time. With monitoring plots, soil samples may be taken every year, which can reflect year-to-year variations much better than sampling every four to six years. This is the recommended temporal interval for grid or targeted sampling [17,25]. However, because monitoring plots do not provide a full indication of field variability, they are not a typical method to derive application maps for liming. Farmers and their advisors still prefer grid or targeted sampling.

With the advent of PA technologies, the demand for high-resolution soil maps becomes much more evident [26] and spatially densed soil sampling becomes sensible (Figure 1D). Thus, efforts have been made to increase sampling density by introducing new technologies. Lütticken reports an automated soil sampling system that carries out preconditioning of soil samples in the field to accelerate conventional laboratory analysis [27]. Another approach to accelerate soil analysis and to reduce cost is to replace standard laboratory methods with new sensors and methods for data processing [26]. In particular, spectrophotometric sensors together with Partial Least-Squares regression offer potentials for high-throughput analysis of soil samples [26,28]. However, these methods still need soil samples brought to the lab and require sample preparation for analysis. The ultimate solution, therefore, would be to conduct soil analysis within the field in real-time.

Any results from new methods for online analyses of soil pH within the field must be comparable with results from standard methods. The standard methods require a number of soil cores to be taken, bulked, and mixed. Then, an aliquot is taken for further analysis in the lab, where it is preconditioned by air drying and sieving. After adding a certain proportion of liquid (distilled water or CaCl_2_ solution) and soaking or shaking, the pH is measured by an ion-selective glass electrode [29]. Sampling procedures, liquids, and liquid ratios may vary between and within countries, but the glass electrode is always used as the standard for pH measurements in soil solutions.

In general, the potentiometric determination of the pH value is defined as follows [30]:

(2) $$\text{pH}(X)=\text{pH}(S)+\frac{E(S)-E(X)}{(R\cdot T/F)\;\text{ln}\;10}$$

where R denotes the gas constant, T the thermodynamic temperature, and F the Faraday constant. E(S) and E(X) describe the electrode potential of a pH cell containing a standard solution with known pH value, pH(S), and a solution with unknown pH value, pH(X). The measuring principle is based on the electrochemical reaction that takes place on the surface of the sensing electrode plunged into the solution of unknown pH. This reaction generates a potential at the sensor electrode, E(X), which is proportional to the logarithm of the hydrogen ion (H^+^) activity in the unknown solution, as described by the Nernst equation [31,32]. While the potential E(S) at the reference electrode is held constant, the pH value is derived from the difference E(S) − E(X).

The glass electrode is the most important electrochemical sensor [31], and it is the standard for soil pH determination in the laboratory [7,29,33]. It uses a thin membrane of sodium silicate glass as the sensing electrode [31]. During the production of the glass membrane, alumina, sodium, and other substances are added to achieve high H^+^ selectivity [31,32]. Thus, these types of electrodes are called ion-selective electrodes (ISE). Because of the fragility of the glass membrane, mechanical strains should be avoided, and it is not advisable to use glass electrodes in harsh environments.

To increase sampling density for pH mapping while keeping costs low, efforts have been made to measure soil pH on-the-go while travelling across the field. Adamchuk *et al.* [33] developed an automated sampling system for measuring soil pH electrochemically based on earlier works [34]. This method takes soil samples automatically from a depth of approximately 10 cm while the device is moving over the field. The soil material is then brought into direct contact with two flat surface combination glass electrodes. The pH value is recorded after stabilization of the electrode output together with GPS coordinates. A subsequent linear regression with reference pH values showed r^2^ of 0.83 and a standard error of prediction of 0.45. Later on, glass electrodes were replaced by antimony electrodes because of the rapid wear and leaching of the glass membrane [35].

Viscarra Rossel *et al.* reported about a soil pH and lime requirement sensing system that consisted of a soil sampler, a sieving unit and an analytical unit [36]. Sieving ensures that only soil material with a particle size of 2 mm or less is passed to the analytical unit, where the soil material is mixed with different extraction solutions (H_2_O, CaCl_2_, Mehlich lime requirement buffer) and analyzed by an ion selective field effect transistor (ISFET). Although a number of problems have yet to be addressed, the initial results are promising. Measurements taken on one field showed average deviations from reference values of 0.37 pH (CaCl_2_) and 0.68 pH (H_2_O). Sethuramasamyraja *et al.* evaluated an Agitated Soil Measurement (ASM) method for integrated on-the-go mapping of soil pH, K and N using ISE [37]. They investigated simultaneous placement of different ISEs into a stirred (agitated) 1:1 soil-water suspension made with deionized water, while washing the electrodes with tap water. They reported high correlations with laboratory reference measurements.

Up to now, only one on-the-go sensing system for soil pH has been commercialized. The system is based on the system developed by Adamchuk *et al.* [33], and was combined with the Veris soil apparent electrical conductivity (ECa) sensor [38]. The so-called pH Manager is now marketed by Veris Technologies (Salina, USA) as a part of the mobile sensor platform Veris MSP. In the US, Adamchuk *et al.* compared the pH maps of the Veris MSP (pH Manager) with maps derived from standard grid sampling on eight fields [39]. They concluded that a field specific calibration was necessary to improve the results obtained with standard grid sampling. In another study [40], the Veris MSP employing antimony ISEs was used on two fields. It was shown that maps derived from on-the-go measurements were more accurate in delineating acidic soil areas than corresponding maps derived from grid sampling or field average methods.

Currently, sensor-based soil pH mapping has already been adopted by agricultural service providers and farmers in the United States (more than 130 units have been sold in the US [41]). However, in Germany and in other European countries, this technique is new. To analyze the possibilities of soil pH on-the-go mapping in Germany, we evaluated the Veris Multi Sensor Platform (MSP) along with its soil pH unit (Soil pH Manager). The main questions addressed in this study were the following:

- How accurate is the sensor? Is its output related to standard determination of soil pH by CaCl_2_ extraction in Germany under laboratory and field conditions?
- Is there a need to calibrate the sensor pH values to obtain a correlation with lab pH values?
- Can soil electrical conductivity, measured along with pH, help to improve calibration?
- What problems may arise when using the Veris Soil pH Manager™ under German soil and farming conditions, and how can we improve the system?
- What is the accuracy of the map obtained from on-the-go measurements compared with maps generated by standard sampling and laboratory analysis, and what are the consequences for lime requirement rates?

## 2. Experimental Section

### 2.1. The Soil pH Manager™

**Figure 2.**
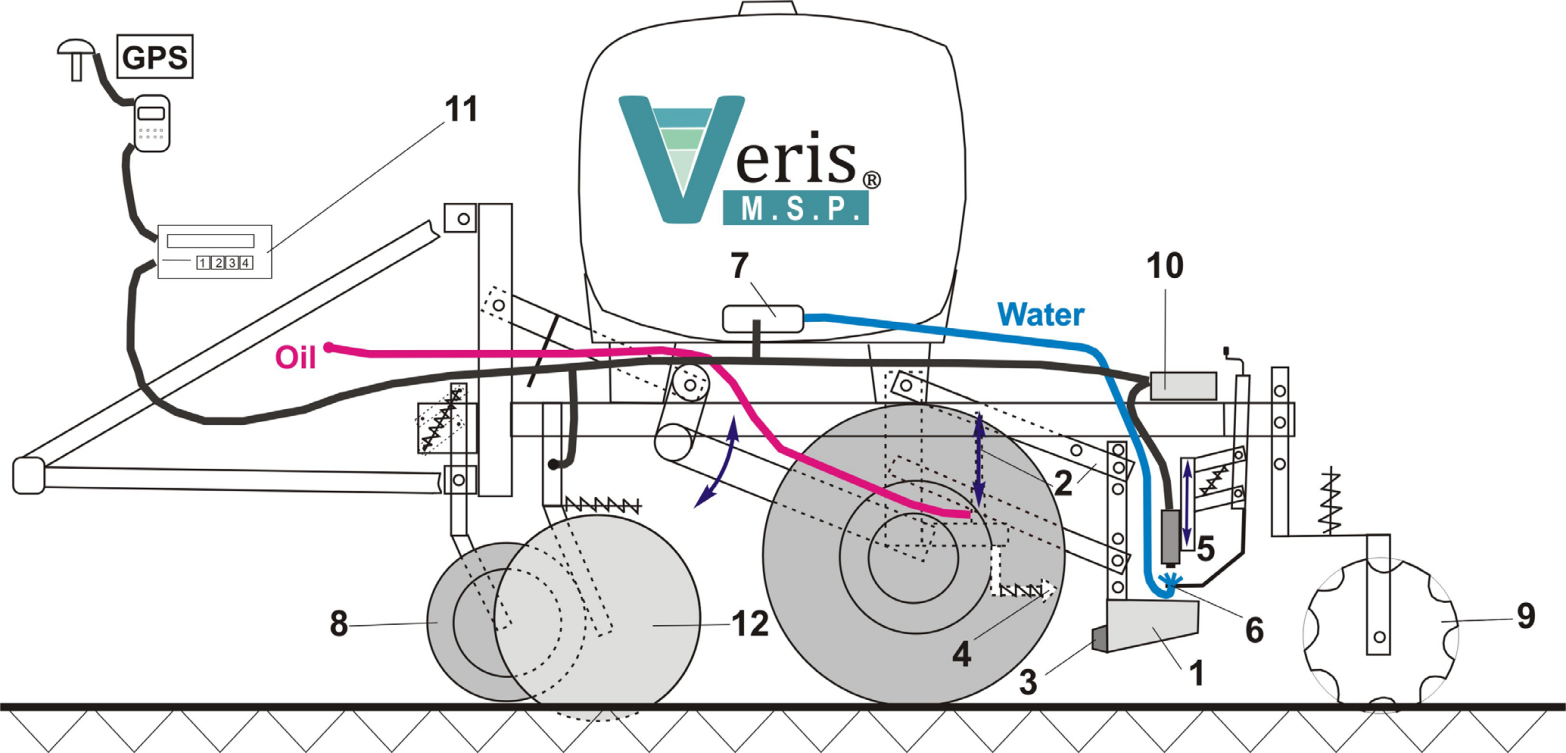
Schematic of the Veris Multi Sensor Platform (MSP) including the Soil pH Manager and the Soil Electrical Conductivity Surveyor. Numbers are explained in the text.

The Soil pH Manager™ of Veris Technologies is a real-time sensor for soil pH mapping. It automatically collects soil samples and measures soil pH from direct contact to the soil material on-the-go. It consists of three main components: a hydraulic soil sampling system, a pH electrode measurement system, and a water wash system (Figure 2). While driving, the soil sampler shoe (1) is lowered into the soil via a hydraulic cylinder on a parallel linkage (2). Sampling depth and time are adjustable but typically set to 0.01 m and 2 s. While in the soil, the front of the shoe cuts the soil material with a cone (3) and produces a soil core flow through the trough of the shoe. Then, the shoe is raised to bring the soil sample into direct contact with two antimony pH electrodes (5). Concurrently, the shoe is passing a scraper (4) to clean the front. The up and downward movement of the shoe is controlled by a proximity sensor. The measurement is done with nontreated, naturally moist soil material, so no solution is added to the soil before being brought into contact with the electrodes. The obtained pH value is then computed from the averaged voltage outputs of the two electrodes. Conversion of voltage to pH units is achieved by a calibration routine that involves measurement of two standard solutions with known pH values of 4 and 7.

Measurement time depends on the electrode response and ranges between 7 and 25 s. After the pH measurement has finished, the shoe is lowered into the soil again and the analyzed soil sample is discharged by the new soil material flowing through the cone. Simultaneously, the electrodes are rinsed by two wash nozzles (6) installed at each side of the electrode holder. The water is stored in a 359 L tank with two electric water pumps (7). Row cleaners (8) remove crop residue in front of the sampler shoe and furrow closers (9) are available to fill the furrow produced by the shoe. The sampling process and the pH electrode signals are managed by an external controller (10), which sends the data to a user interaction device (11). It is possible to set the controller to manual state, which allows manual operation of the sampler and the water wash system. During field operation, differential GPS coordinates are recorded at the moment the sampler shoe is pulled out of the soil.

The Soil pH Manager™ is a module of the Veris Mobile Sensor Platform (MSP), which consists of a soil electrical conductivity/resistivity device as the base unit (12). The apparent electrical resistivity (Ω·m) is measured by the four-point method and converted to apparent electrical conductivity (ECa in mS·m^−1^). Six EC coulter electrodes (rolling electrodes) enable the system to map soil ECa data continuously from two soil depths. The effective depth (depth above which 50% of signal contribution effect is derived) for the narrow electrodes is 0.12 m (ECa_shallow_), and 0.37 m for the wide electrodes (ECa_deep_) (see [42] for further details).

### 2.2. Electrochemical pH Measurement Using Antimony Electrodes

**Figure 3.**
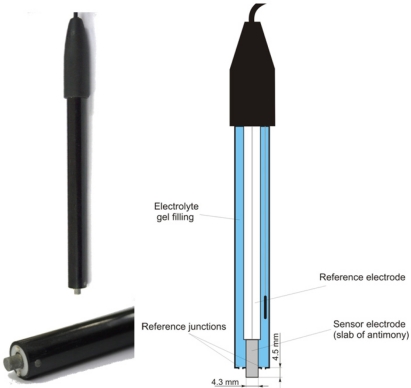
The antimony electrode used with the Soil pH Manager (length: 120 mm, diameter: 12 mm).

The soil pH sensor uses antimony electrodes (Figure 3) instead of glass electrodes because of their greater robustness [35]. The assembly of the antimony electrode is very similar to the glass electrode but consists of a slab of antimony as the sensor electrode. On its surface, an adsorbed film of mainly antimony trioxides (Sb_2_O_3_) is present, which is formed by air oxidation. The potential of the sensor electrode is the consequence of a reaction of this adsorbed film with H^+^ ions in the solution [43]:

(3) $${\mathrm{Sb}}_{2}O_{3}+6H^{+}+6e=2\mathrm{Sb}+3H_{2}O$$

As can be derived from Equation (2), the resulting potential, E_e_, changes linearly with pH [43]:

(4) $$E_{e}=E_{e}(0)-\frac{R\cdot T\cdot \;\text{ln}\;10}{F}\mathrm{pH}(X)$$

Despite the obvious similarities, there are some marked differences of antimony electrodes when compared with glass electrodes. Generally, the initial electrode potential E_e_(0) is lower than glass electrodes. Furthermore, the resulting electrode potential, E_e_, is influenced by several oxidizing and reducing substances that affect the Sb species at the electrode surface, such as dissolved oxygen, carbon dioxide or hydroxyacids [44]. Cations replaceable by antimony cause a disturbance when present in the solution and phosphate is known to have an effect on the potential [45]. Additionally, the potential of the electrode can also be altered by stirring the solution [43]. Thus, the potentials of individual antimony electrodes are deemed to be poorly reproducible, and the observed response characteristics may deviate from theoretical assumptions [43]. However, several studies found good agreement between antimony electrodes and glass electrodes for pH measurement in soil solutions [44,46]. The main advantage of the antimony electrode is its robustness. Mechanical cleaning of the electrode’s surface is considered as uncritical [47].

For accurate measurements, it is essential to calibrate the antimony electrode with several buffer solutions of known pH value [43]. Therefore, we calibrated the Veris antimony electrodes with a two-point calibration using standard buffer solutions of pH 4 and 7 at the beginning of each experiment. We later refer to this as the basic calibration. At the beginning of the calibration, the electrodes were inserted into the buffer solution and the solution was gently stirred for 30 s to achieve a steady state. Both electrodes were simultaneously calibrated using the protocol of the Veris instrument.

### 2.3. Laboratory Reference Measurements

All soil samples were analyzed for soil pH following the German standard protocol for the determination of lime requirements [48]. Soil samples were oven dried (40 °C) and sieved (2 mm). Then, 10 g of soil material was mixed with 25 mL 0.01 M CaCl_2_ solution. The soil suspension was shaken for 1 h and stored without agitation for 1 h. Before the pH measurement, the soil suspension was shaken again. The pH measurement was conducted using a glass electrode, which was calibrated using a two-point buffer standard (pH 4 and 7). The electrode reading was considered stable when no change of more than 0.02 pH occurred within 5 s.

Soil pH (CaCl_2_) is the most important parameter for calculation lime requirements in Germany [48]. The use of CaCl_2_ solution for pH determination is preferred in plant nutrition and soil science because it minimizes the dilution and paste effects observed with pH measurements in pure soil/water solutions [29]. However, in addition to the activity of solute H^+^, a small amount of reserve H^+^ acidity is measured due to cation exchange by Ca^2+^.

### 2.4. Experimental Design

For adoption of a new system, it is important to know what quality it provides against an established standard. A deviation of less than ±0.05 pH units would be the ultimate goal because such an error will not cause any changes when determining lime requirement (see Section 2.5). Experiments with the soil pH sensor were therefore conducted under different conditions: (a) controlled conditions in the lab, (b) semicontrolled conditions moving the sensor in a stop-and-go mode along a transect, and (c) practical conditions working in the regular on-the-go mode.

Experiments (a) and (b) mainly addressed issues related to the differences between measurements with the standard glass electrode in a soil solution and the antimony electrode of the pH Manager in field moist soil. Tests under practical conditions (c) addressed problems invoked by on-the-go field operation, the advantages of the soil pH sensor compared with conventional mapping, and its utility to derive application maps for liming.

Outdoor measurements were conducted in three fields (A, B, and C). All fields were located in Northeastern Germany. Table 1 comprises some field properties. In Northeastern Germany, soils were formed on glacial and periglacial sediments. Most topsoils are acid and sandy. However, pH values can vary due to the influence of carbonates on soils formed on glacial till.

#### Experiments under Controlled Conditions

The first experiment tested the pH measurement system of the soil pH sensor under laboratory conditions. Four preselected soil samples (400 g) that spanned the pH range between 4 and 7 were measured repeatedly with direct contact of the two antimony electrodes using the Veris data logger as the measurement instrument. To assure homogeneity and the same level of moisture for each sample, soil material was grounded, sieved (2 mm) and oven dried (40 °C) before 10% deionized water was added to the soil sample. Before each measurement, the electrodes were washed for 10 s with deionized water and then were gently pressed into the soil material until a complete coverage of the tip of the electrode was guaranteed (approx. 10 mm). In the first test, the electrode’s response time was analyzed by taking sensor readings after 5, 10 and 20 s. Measurements were then compared with laboratory reference measurements [48]. All measurements were repeated 10 times. In a second test, the antimony electrode was analyzed for dependence of subsequent measurements on previous measurements. To reveal possible “memory effects” of antimony electrodes two soil samples of pH 4 and 7 were measured in an alternating sequence.

#### Experiments under Semicontrolled Conditions

In this experiment, the soil pH sensor was brought outside (field C) and operated in the manual mode. On two transects (330 and 440 m), 55 measurements were taken and measured with antimony and glass electrodes. While moving the system with a tractor, the soil sampler shoe was lowered manually into the soil and raised again after a soil core flow through the trough had been developed. The tractor was stopped, and the pH readings were noted after the electrodes had been pressed against the soil material for 15 s. After that, the electrodes were washed with deionized water using the two wash nozzles and were dried with a piece of paper. Soil material within the trough was collected for reference measurement with glass electrodes in the lab. Then, the tractor pulled the sensor platform to the next measurement point while the shoe was lowered into the soil. At the start of each transect and in the middle of transect two, both electrodes were calibrated using the basic calibration procedure described above. This was done in order to account for possible temperature drift and other ambient influences.

#### Experiments under Practical Conditions

Field tests were conducted in fields A, B, and C. Before each field measurement, the electrodes were calibrated as described above. Sampling and measuring was conducted automatically in the typical on-the-go mode and electrodes were washed using tap water (7.6 pH and 1,060 μm cm^−1^). While sampling, soil ECa measurements were recorded continuously along the track. Soil samples were collected one day after each field was measured. These samples were taken from the soil material remaining in the furrows caused by the soil sampler. The sampling and measurement positions were located by GPS using Trimble AgGPS with Omnistar differential correction signal (±1 m accuracy). Samples were analyzed for soil pH in the laboratory by the standard method described above. The lab values were used for accuracy assessment and field-specific calibration. This was done by linear regression analysis (see Section 2.6). The squared correlation coefficient r^2^ was used to qualify the random error, while the intercept and the slope of the regression model were indicators of systematic errors and electrode sensitivities, respectively. Additionally, the mean absolute errors (MA) and the mean error (ME) were calculated to summarize the magnitude of the errors. The linear regression models were additionally used for field-specific calibrations in order to transform Veris on-the-go field measurements into pH (CaCl_2_). Establishing a relationship between on-the-go pH and pH (CaCl_2_) is highly relevant because pH (CaCl_2_) values are used as the main input for calculating lime requirements (see next section).

### 2.5. Benefits of the on-The-Go Approach Compared with Standard Protocols

#### Comparison of Mapping Strategies

To evaluate the potential of on-the-go mapping quantitatively, two maps employing different standard sampling strategies were compared with on-the-go measurements from field A. Therefore, soil was collected at 55 locations spatially separated from the furrows formed by the sampler of the Veris pH Manager. These samples will be referred to as standard samples. Each standard sample was composed of 16 auger cores at a sampling depth of 0.25 m and distributed randomly within a circle 1 m in diameter. Sample locations were irregular distributed over the field. All standard samples were analyzed for soil pH in the laboratory.

The first standard map (map_WF_) simulated the results of a whole field sampling strategy. Nine standard soil samples were randomly chosen to cover the range of pH values and to achieve a regular distribution of locations within the field. The pH values of these samples were then averaged to obtain a uniform soil pH value, which was attributed to the entire field area to form the first standard map.

The second standard map (map_AC_) simulated the results of an area composite sampling strategy. For this purpose, the field was equally divided into three zones of approximately 1 ha. In each zone, three standard soil samples were averaged, and the averaged value was attributed to that zone. For the on-the-go map (map_onthego_), the on-the-go pH measurements were calibrated and interpolated using the procedures below. The remaining 46 standard samples were used for validation.

#### Consequences for Liming

Liming needs were computed using German guidelines for fertilization (Table 2, [49]). This algorithm requires pH (CaCl_2_), soil texture and soil organic matter content as inputs. Organic matter content in the soils of field A was below 4% and, thus, did not induce any variation in lime recommendation. Soil textural variation was low, and was assumed to be uniform throughout the field A. The differences between the lime requirement of the maps and the lime requirement of the validation samples were then extrapolated to the entire field.

### 2.6. Data Analysis

To quantify the precision and accuracy of the soil pH sensor, the sensor readings (*pH_OTG_*) were compared with their corresponding lab reference values (*pH_REF_*). The relationship between *pH_OTG_* and *pH_REF_* was then evaluated using linear regression:

(5) $${\mathrm{pH}}_{\mathrm{OTG}}=a+b\cdot {\mathrm{pH}}_{\mathrm{REF}}+\varepsilon$$

Here, *a* and *b* denote the intercept and slope of the linear model found by minimizing the error (*ε*) between *pH_OTG_* and *pH_REF_* using the least squares approach. As shown previously [35], on-the-go pH values may systematically deviate from lab pH values from field to field. This makes a calibration necessary with field-specific calibration samples in addition to the basic calibration of the electrodes using standard pH buffer solutions. For field-specific calibration, 10 samples were chosen from each of the soil samples collected on fields A, B, and C as calibration samples (*pH_CAL_*). Selection criteria were pH range and regular distribution within the field. The remaining soil samples were used for validation (*pH_VAL_*). A linear model can be estimated from the given calibration sample values and their corresponding on-the-go measurements:

(6) $${\mathrm{pH}}_{\mathrm{OTG}}=a+b\cdot {\mathrm{pH}}_{\mathrm{CAL}}+\varepsilon$$

The inverse estimator is given by the simple linear regression of *pH_CAL_* on *pH_OTG_* [50]:

(7) $${\mathrm{pH}}_{\mathrm{CAL}}=a_{\mathrm{CAL}}+b_{\mathrm{CAL}}\cdot {\mathrm{pH}}_{\mathrm{OTG}}$$

The calibration parameters *a_CAL_* and *b_CAL_* are estimated and allow the linear transformation of the uncalibrated *pH_OTG_* values. This calibration model can be extended by including co-variables. In our case, we tested if the integration of soil ECa could improve the results of the calibration model. A much simpler calibration was proposed by [35], who suggested field-specific calibration by only shifting the *pH_OTG_* values. This was implemented here by using the mean difference between *pH_OTG_* and *pH_CAL_* as the shifting parameter.

The quality of the calibration model was evaluated by comparing the mean absolute error (MAE) between the pH_OTG_ values and the pH_VAL_ values before and after the calibration:

(8) $${\mathrm{MAE}}_{\mathrm{raw}}=\frac{1}{n}\sum_{i=1}^{n}\left|{\mathrm{pH}}_{\mathrm{VAL}}-{\mathrm{pH}}_{\mathrm{OTGraw}}\right|$$

(9) $${\mathrm{MAE}}_{\mathrm{cal}}=\frac{1}{n}\sum_{i=1}^{n}\left|{\mathrm{pH}}_{\mathrm{VAL}}-{\mathrm{pH}}_{\mathrm{OTGcal}}\right|$$

Geostatistical methods were used to analyze the spatial structure and to compute spatial maps of the soil pH measurements. The omnidirectional experimental semivariogram *γ̂(h)* showed the average dissimilarity between the observations, *z(x)*, as a function of separation distance, *h*, with *n(h)* denoting the number of squared differences of pH values at the locations *x_i_* and *x_i_* *+ h* separated by the distance vector, *h* [51]:

(10) $$\hat{\gamma }(h)=\frac{1}{2n(h)}\sum_{i=1}^{n}{\left(z(x_{i})-z(x_{i}+h)\right)}^{2}$$

We also computed directional experimental semivariograms that included the relative direction between the point pairs (*i.e.*, only point pairs that fell into a given angular tolerance were classified within the lag classes of the experimental semivariogram). The omnidirectional experimental semivariograms were then parameterized using the spherical semivariogram model [51]. Parameters were computed from the semivariogram model as follows: The “range” quantified the distance within which observations were statistically dependent (autocorrelated). The “partial sill” denoted the structured variance that was explained by neighboring samples. The nugget effect enumerated the discontinuity of the semivariogram model at its origin (*h* = 0). The nugget effect was composed of the unexplained variation that could not be resolved by the given sampling resolution (microvariance) and the sample measurement error. The “nugget to sill ratio” (NSR) then expressed the percentage of the nugget effect on the total sill (nugget effect + partial sill). Soil pH maps were computed using ordinary kriging, or, if a spatial trend was present, by universal kriging [51].

## 3. Results and Discussion

### 3.1. Testing the Soil Sensor under Controlled Conditions

**Figure 4.**
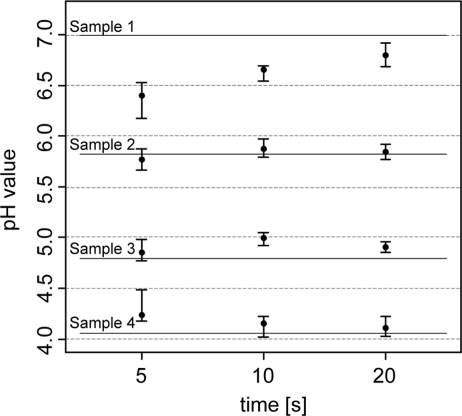
Soil pH measurements using antimony electrodes at 5, 10 and 20 s time intervals compared with lab reference measurements under laboratory conditions. Points correspond to the median of 10 replicates. The whiskers of the box plots display the range of the repeated measurements. The thick lines show the corresponding lab pH value.

In the analysis of the response time of the antimony electrodes, the pH readings became more stable the longer the electrodes were retained within the soil (Figure 4). After 5 s of retention, pH values varied between 0.20 and 0.35, and after 20 s, they fluctuated within 0.10 and 0.25 pH units. Laboratory pH measurements (lab pH) using the standard protocol of pH determination were much more precise.

Lab pH only varied between 0.02 and 0.07 pH units. In terms of measurement accuracy, sensor pH readings differed from the lab pH by 0.22 pH units (5 s) and 0.09 pH (20 s) units. Other studies found similar or greater differences. Conkling and Blanchar [44] compared an antimony electrode with a glass electrode in a soil-water solution and showed an average discrepancy of 0.32 pH units, while Baghdady [46] observed a difference of only 0.07 pH units when measuring a soil buffer solution. In our test, discrepancies between antimony and glass electrodes seemed to depend on the pH value. As the pH values became more extreme (pH 4 and 7), the antimony electrode readings showed an increasing offset compared with the lab pH. With lower lab pH, antimony electrode readings became higher, while they were lower with higher lab pH. These differences were more pronounced with the 5 sec measurements. The discrepancies can be explained by the direct contact soil measurement principle of the Veris sensor. On the contrary, the standard laboratory method requires mixing of the soil material with the CaCl_2_ solution. This will replace H^+^ ions on soil particles and the analyzed soil solution will hold a higher H^+^ activity than in field moist soil. Also, the short measurement time of the sensor has an influence on its accuracy. Measurement time was restricted to about 5–20 s during the field experiments. In the laboratory, the pH measurement value is taken after the electrode potential has stabilized. Antimony electrodes require several minutes to obtain a practical constant potential difference [52]. In addition, the electrode readings will be influenced by the sensor electronics and other specific properties of the antimony electrode. In particular, some studies indicated an underestimation of high soil pH values [35,44] and an overestimation of low pH values by using antimony electrodes [53]. Additionally, the strong deviations of the sensor readings in soil sample A (with the high pH) may have also been affected by inhomogeneities within the sample known as the ‘nugget effect’ or micro-variability [51]. We detected small particles of carbonate, which is typical for calcic soils in northern Europe formed on glacial till.

**Figure 5.**
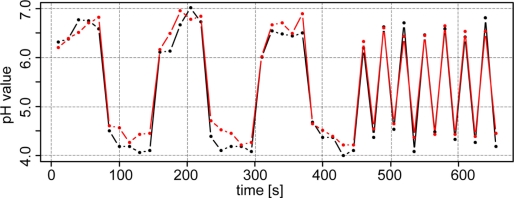
Consecutive measurements with the pH antimony electrode 1 (black) and 2 (red) in two soil samples with different pH values under laboratory conditions.

To analyze whether the antimony electrodes were exhibiting a memory effect, two soil samples with pH 4 and 7 were measured in sequence. In fact, a memory effect was observed, as depicted in Figure 5 (left). Depending on whether the previous sample had a lower or higher pH, repeated pH readings from the proceeding sample were slowly increased or decreased, respectively. This memory effect was also evident when measuring alternately from sample to sample (Figure 5, right). In this case, the antimony electrodes were unable to capture the full range of the pH values. Therefore, when soil pH is changing rapidly, the antimony electrodes will slightly underestimate or overestimate the pH value depending on the previous sample.

### 3.2. Testing the Soil Sensor under Semicontrolled Conditions

**Figure 6.**
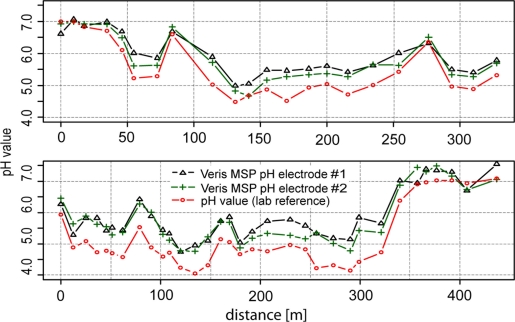
Measurements on two transects with manual control of Veris MSP antimony pH electrodes compared with measurements with glass electrodes in the lab. Soil material was identical for both types of measurements.

Figure 6 shows the results of experiments under semicontrolled conditions on a transect. Within 400 m, high differences of soil pH were observed ranging from approximately 4 to 7.5 pH (lab), highlighting the necessity of high-resolution mapping of soil pH. With a few exceptions, the line graphs of the two antimony electrodes ran parallel to the line of the glass electrode. In general, readings of electrode #2 were slightly lower than electrode #1 (−0.11 pH), which may have been due to the specific properties of each electrode. Additionally, more pronounced differences occurred in the second section of transect two after recalibration of the electrodes. Fluctuations between the two electrodes may have also been due to the heterogeneous nature of the soil in the trough.

As we observed for the test under controlled conditions in the lab, the soil pH sensor readings were higher than pH (CaCl_2_) within soils with lower soil pH (−0.5 pH) values. This systematic effect was stronger in this test than in the laboratory experiment. Measurements in soils with pH values lower than 6 were increased by 0.65 pH units and measurements in soils with pH values higher than 6 were increased by just 0.19 pH units compared with lab pH (CaCl_2_).

**Figure 7.**
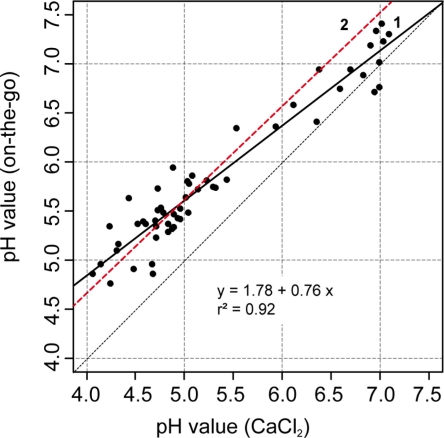
Scatter plot of on-the-go measurements and lab pH values with the corresponding regression model (black line, 1) from transects shown in Figure 6. The black dotted line shows the 1:1 relationship and the red dotted line (2) is the regression model of [56].

The relation between sensor readings and lab reference is further illustrated in the scatter plot shown in Figure 7. The data was accurately described by a linear regression model using lab pH as the independent variable (r^2^ = 0.92). In addition, the regression line clearly deviated from the 1:1 line, underpinning the systematic error we observed in this experiment. We explained this deviation mainly by the effect of the CaCl_2_ solution as an extractant even though temperature variability and characteristics of the sensor electronics might have contributed as well. For example, Conyer and Blanchar [44] analyzed the correlation between pH measured in a CaCl_2_ solution and in a H_2_O/soil dispersion using a glass electrode. The resulting linear relationship between the CaCl_2_ and H_2_O solutions took the form pH_CaCl_ = 1.05 pH_w_ − 0.9, to which our regression parameters were a close fit. Furthermore, later studies that analyzed a greater number of different soil samples revealed curvilinear relationships between pH measurements with CaCl_2_ and H_2_O solutions [54,55]. In our case, the scatter plot did not show nonlinear behavior. However, the intercept was approximately 1.8 pH units and the steepness of the slope of the regression line was less than the 1:1 line in the scatter plot, leading to a high mean absolute error of 0.55 pH units and a strong bias (mean error = −0.53). Thus, calibration was necessary to reduce the deviation between the soil pH sensor readings and pH (CaCl_2_).

Among other factors, like sample inhomogeneity and temperature variability, field tests may be affected by spatial variations of soil water content. This might happen in at least two ways: first, increasing water content may decrease H^+^ concentration due to a dilution effect; and second, increasing water content may raise pH readings, as explained by the Debye-Hückel theory. These are contrary effects and the second one requires some explanation. High concentrations of ions can lead to a deviation from the theoretical linear relationship between the concentration of ions in the solution and the electrical potential [32]. This is due to the fact that ion activity, which is the “effective concentration” in terms of the law of mass action [32], is reduced when the concentration of ions rises. With increasing concentration, ions start influencing each other and consequently reduce their mobility and reaction rates. The relationship of activity and concentration is then described by the Debye-Hückel theory [32].

In typical well-drained soils, the total concentration of ions in the water varies between 0.001 and 0.01 mol L^−1^ [57]. According to the Debye-Hückel theory, this range of concentrations can have some effect on ion activity. Therefore, Adamchuk *et al.* [33] analyzed the output of glass electrodes depending on soil water content. They observed a slight increase in pH readings when water was added to the soil, but repeated measurements with the same moisture content showed high variability. Thus, they concluded that there was no significant effect within the range of moisture contents typical for soils in the field. However, rinsing the electrodes with wash water may also indirectly affect the ion concentration. In our experiments, 0.10 mL of wash water was adhered at the sensor electrode on average, which is a very small amount and the effect may be neglible.

In addition, temperature variability can influence pH measurements in at least three ways. Firstly, potentiometric measurements are related to temperature as given by Equations 2 and 4. Second, dissociation of molecules into protons and bases is a function of temperature. Third, the electronics of the pH instrument can be affected. In our case, field measurements of soil temperature showed variations of less than 5 K. Thus, soil temperature had no relevant effect on random errors but could have contributed to systematic errors. The sensitivity of the Veris pH instrument was not fully explored in this study and should be further analyzed in subsequent experiments.

### 3.3. Testing the Soil Sensor under Field Conditions

**Figure 8.**
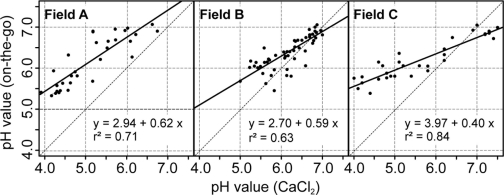
Scatter plots showing the relationship between on-the-go pH measurements and corresponding lab values on three fields. Soil samples for both analyses were taken from nearly identical locations.

The use of the soil pH sensor on fields A, B and C resulted in high measurement densities of 91.1, 26.54 and 34.4 samples ha^−1^. The measurement density depended mainly on the speed and the spacing between the tracks. This represents a great improvement over manual standard sampling for site specific management practices in Germany that normally relies on 3 ha sample zones. To make full use of the on-the-go measurements, it is important to establish calibration models that convert the sensor readings into pH (CaCl_2_) which is the main input for calculating lime requirements. Differences of 0.1 pH (CaCl_2_) units amount in differences in lime requirements of 300 to 400 kg CaO per hectare on sandy soils (Table 2). In Figure 8, scatter plots depict the relationship between the on-the-go measurements and the lab pH values given by the soil samples collected within the furrows. On all fields, the raw, uncalibrated on-the-go measurements were linearly well correlated with the lab reference values, and r^2^ values ranged between 0.63 and 0.84. These r^2^ values were slightly smaller compared with r^2^ values obtained by studies conducted in the United States, which were mainly between 0.73 and 0.97 [39,40,58]. A similar study conducted in Germany reported an r^2^ value of about 0.62 computed over multiple fields [59].

However, a systematic deviation was observed with the on-the-go measurements and intercept values ranged between pH 2.7 and 3.97. In this case, the intercept values were even larger and the slope values were smaller compared with the field transect test of the previous section. In addition, the raw on-the-go measurements below 4.5 pH strongly deviated from the lab reference. This may have resulted from the relatively short measurement time of approximately 5–20 s during the field tests. To reduce this deviation, a field-specific calibration of the on-the-go measurements was conducted based on 10 calibration samples using lab reference values with inverse regression (Table 3). All resulting linear models were significant (p < 0.01) and the field-specific calibration had a strong effect on the errors between on-the-go measurements and lab reference values. The mean absolute errors were therefore reduced for fields A, B, and C by −0.79, −0.04 and −0.37 pH units, respectively. The relatively small improvement on field B was due to the overall small variability of soil pH in this field. Furthermore, the bias, in terms of the mean error, was greatly reduced.

Adamchuk *et al.* concluded that adding a constant value to the on-the-go measurements was sufficient for calibration [39]. However, in our case, this simple shifting of values was not adequate. It improved the results only for field A and B, but not for field C. Therefore, we conclude that the use of the pH (CaCl_2_) method as the reference demands the two parameter linear regression model for field-specific calibration of the on-the-go measurements.

Soil ECa and on-the-go measurements corresponded reasonably well on field A and C. Best correlations, were found for field A with ECa_shallow_(0.57) and for field C with ECa_deep_(−0.56). On field B, no significant correlation with ECa was found. The integration of soil ECa into the field-specific calibration model improved results only for field C. For this field, the MAE decreased from 0.45 to 0.40 pH units using soil ECa_deep_. Soil variability in field C was strongly influenced by geology. Soil texture varied greatly and lime material was found on the hilltops. In this field, spatial changes in the reserve acidity may force pH values measured with CaCl_2_ in the lab to differ from measurements of the active acidity directly in the soil because the latter method does not extract H^+^ adsorbed onto the exchange complex in soil colloids. Thus, soil ECa measurements helped to explain the relationship between both methods.

**Figure 9.**
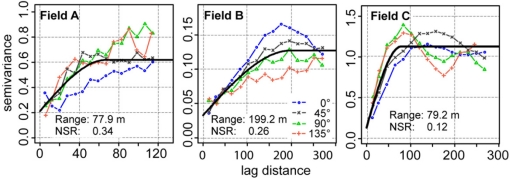
Directional experimental semivariograms of the calibrated on-the-go pH measurements computed in four different directions. The 0° indicates the direction along the measurement track. The black line denotes the omnidirectional semivariogram model.

The high sample density provided by the on-the-go sensor made it possible to compute reliable semivariograms in multiple directions (Figure 9). The semivariogram values revealed a short-range variability in field A and a long-range variability in fields B and C. Except for field B, semivariogram values were smaller in the direction along the track of the measurement. In this direction, measurement values were more connected with each other. This may have been the results of an inter-correlation of measurement errors occurring along track.

All semivariogram models had NSR values greater than zero. The percentage of the nugget effect on the semivariogram model ranged between 26 and 34%. The error component of the nugget effect could then be estimated from the residuals of the linear model when regressing the calibrated on-the-go measurements on the lab reference. This showed that the measurement error greatly influenced the nugget effect, with percentages of 49, 68 and 30% for fields A, B, and C, respectively.

During the field measurements we encountered several types of problems. For example, the soil sampler shoe was blocked by residues and roots of weeds, so that no soil flow was produced through the trough of the shoe. This was observed quite often in field A because the roots of couch grass were accumulated at the front side of the shoe and clogged the entrance. In other cases, the sampler shoe was not correctly within the soil and no new soil material could flow into the trough. Also, the wash nozzles were sometimes displaced by accumulated residues so the electrodes were not washed sufficiently. These mechanical problems caused soil material to be relocated from one point to the other, nonsoil material, such as plant residues, to be measured, and the electrodes to be inadequately cleaned.

Also, it must be mentioned that the soil material is not sufficiently mixed and only soil material along a small furrow is brought upwards to the electrode for measurement. In standard sampling a greater area is covered by several aliquots and the soil material is mixed thoroughly before lab analysis. This reduced sample support can become problematic, when the pH value varies strongly within small distances because the relevant information is not sufficiently extracted from the spatial pattern of soil pH in the field [60]. To these sampling and mechanical issues, the fact must be added that the soil pH, obtained with the on-the-go sensor, were systematically higher compared with the lab reference and an additional field-specific calibration was necessary to reduce the influences of the sensor electronics and the direct measurement in the soil.

**Figure 10.**
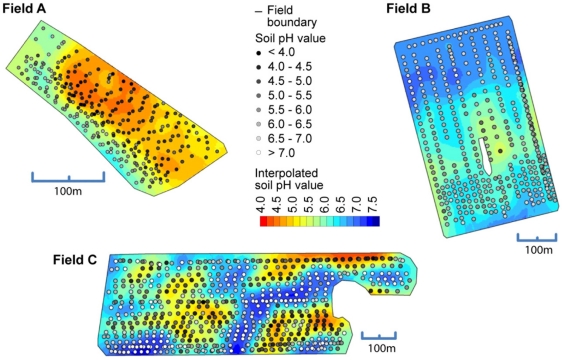
Soil pH maps derived from on-the-go sensor readings after calibration to pH (CaCl_2_). Dots, indicating the measurement locations, are superimposed on raster maps obtained by Kriging interpolation.

Figure 10 shows the spatial maps computed with Kriging using the on-the-go measurements. All maps showed agreeable surfaces and measurement errors seemed not to produce too many awkward features in the maps. The maps also reflected how sudden changes in soil pH can occur in the fields.

### 3.4. Benefit of On-The-Go Mapping Compared with Standard Practical Sampling

**Figure 11.**
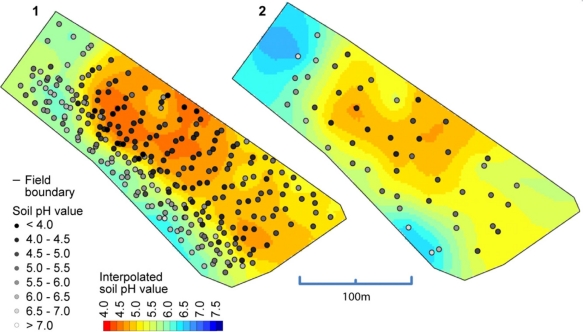
Soil pH map of field A computed from calibrated on-the-go sensor readings (1) and soil pH map computed from standard sampling and lab analysis (2).

So far, we have only compared the on-the-go measurements with the soil samples that were collected from (approximately) identical locations. But what accuracy can we expect when validated against samples that were not co-located? In Figure 11, a map computed from the on-the-go measurements and a map computed from all of the standard samples is depicted. Between these maps, there is a high degree of similarity, especially for the zone with low pH values in the middle of the map. Clearly, the left map shows much more detail due to the greater amount of sample points available with on-the-go soil mapping, but one must be aware that some of the features shown in this map can be the result of measurement errors. The observed overall correlation between both maps computed on a grid cell basis was 0.86 (Pearson’s r).

Table 4 compares the accuracy from on-the-go soil pH mapping with the accuracy of standard sampling strategies (whole field (WF) sampling and area composite (AC) sampling). All sampling strategies showed mean errors unequal to zero. WF and AC sampling inherited this bias due to the coarse sampling, and the on-the-go mapping results were biased because of the differences between direct soil measurement and lab measurement. On average, on-the-go measurements reduced the error by 0.24 and 0.27 pH units compared with AC and WF sampling, respectively. Surprisingly, the MAE was only slightly increased (0.05 pH units) in comparison with the validation of the reference samples above, although the validation samples were taken spatially separated from the sampling positions and up to a soil depth of 0.25 m. Additionally, the measurement accuracy on this field was well in line with results obtained by [39], who reported MAEs between 0.17 and 0.44 pH units.

The observed higher accuracy also positively affected the lime recommendation. In total, the absolute error in calculated lime rates was approximately twice as high with WF and AC sampling compared with on-the go mapping because of its more accurate delineation of soil pH areas. However, the bias was not completely removed by the field-specific calibration and on-the-go mapping estimated a slight over-application of lime for this field. WF sampling, on the other hand, misinterpreted low soil pH areas and would have resulted in an under-application of lime for this field.

## 4. Conclusions

Soil pH mapping using an on-the-go soil pH sensor was successfully carried out on three fields in Northeastern Germany. Preceding tests under controlled conditions demonstrated a high degree of linear relationship between standard laboratory soil pH values and sensor pH values. However, these tests also showed that additional calibration is necessary to reduce errors when predicting pH (CaCl_2_). This is of importance because differences in soil pH of 0.1 units can lead to differences in lime recommendations of up to 400 kg·ha^−1^ CaO.

Under practical field conditions, soil pH values obtained with the soil pH sensor were well correlated with lab pH (CaCl_2_) values. Calibration using 10 reference samples greatly reduced the error between lab pH (CaCl_2_) and sensor pH. The inclusion of the soil ECa data recorded along with the soil pH measurements further improved the calibration on one field with high texture variations. After the calibration, mean absolute errors ranged from 0.28 to 0.40 pH units (CaCl_2_). Some of the erroneous measurements were due to mechanical problems that led to blocking of the system by residues and weed roots. To limit these problems, it would be sensible to install a video camera to monitor the sampling process. The driver would then be able to instantly take care of this type of problem. For improving postprocessing, the driver should be able to set a flag at the measurement point where the problem had occurred. Even with these improvements, it can be assumed that individual measurements of an automatic system in the field will be less accurate than pH values derived from soil analysis in the lab. An on-the-go analysis will only allow for limited control during the pH measurement.

This uncertainty, however, did not ostensibly influence the maps generated by the sensor pH values. Our maps showed highly agreeable patterns of soil pH. The comparison with standard maps demonstrated that map accuracy was improved and the error in calculating lime requirement was reduced using soil pH on-the-go mapping over standard sampling approaches. This was because the higher uncertainty of individual measurements can easily be compensated by the higher sampling density compared with conventional methods [12]. This makes the on-the-go sensor investigated here an interesting alternative for spatially dense sampling strategies when determining the soil pH variability within the field. Further investigations are necessary to improve the calibration function using additional sensor information as co-variables, such as soil reflectance, soil temperature, and soil humidity.

## Figures and Tables

**Table 1.** Field conditions.

	**Soil texture**	**Soil pH range pH(CaCl_2_)**	**Geology**	**Date of measurement**
Field A	Sand/Silty sand	3.83–6.75	Fluvial deposits	23.03.2010
Field B	Silty sand	5.00–7.00	Glaciofluvial deposits (outwash)	15.04.2010
Field C	Loam/silty sand	4.0–7.5	Glacial deposits	05.08.2010

**Table 2.** German recommendation table for liming (kg ha^−1^). Excerpt from [49] showing recommendation only for soil pH values within the range of 4.5 to 5.5.

**Soil pH**	**Sand**	**Weakly loamy sand**	**Strongly loamy sand**	**Silty loam**	**Clay loam/clay**
…	…	…	…	…	…
4.5	3,000	5,700	8,700	11,700	16,000
4.6	2,700	5,300	8,200	11,100	15,200
4.7	2,400	4,900	7,700	10,500	14,400
4.8	2,200	4,600	7,200	10,000	13,600
4.9	1,900	4,200	6,700	9,400	12,800
5	1,600	3,800	6,300	8,800	12,100
5.1	1,300	3,400	5,800	8,200	11,300
5.2	1,000	3,000	5,300	7,600	10,500
5.3	700	2,600	4,900	7,000	9,800
5.4	600	2,200	4,400	6,500	9,000
5.5	600	1,900	3,900	5,900	8,200
…	…	…	…	…	…

**Table 3.** Regression coefficients of the inverse regression between calibration pH(CaCl_2_) and on-the-go pH, mean absolute error (MAE), and mean error (ME) before and **^§^**after calibration.

**Field**	**Intercept**	**Slope**	**Mean shift**	**p-value**	**MAE**	**ME**	**MAE^§^**	**ME^§^**
A	−1.99	1.16***	−0.99	0.0008	1.09	1.08	0.30/0.31^#^	0.10/0.10^#^
B	−2.51	1.36***	−0.25	0.0006	0.32	0.20	0.28/0.28^#^	0.04/0.05^#^
C	−5.89*	1.89***	−0.47	0.0007	0.82	0.52	0.45/0.72^#^	0.11/0.05^#^

‘***’ (α = 0.001)

‘**’ (α = 0.01)

‘*’ (α = 0.05)

‘#’ shifted

**Table 4.** Comparison of whole field sampling with on-the-go mapping.

**Sample strategy**	**MAE (pH)**	**ME (pH)**	**Over (+) or under (−) application of lime in kg**
map_wf_	0.62	−0.15	−3,643
map_ac_	0.59	−0.11	−2,855
map_onthego_	0.35	0.20	+1,394
